# Production and statistical optimization of Paromomycin by *Streptomyces rimosus* NRRL 2455 in solid state fermentation

**DOI:** 10.1186/s12866-021-02093-6

**Published:** 2021-01-23

**Authors:** Ghadir S. El-Housseiny, Asmaa A. Ibrahim, Mahmoud A. Yassien, Khaled M. Aboshanab

**Affiliations:** Department of Microbiology and Immunology, Faculty of Pharmacy, Ain Shams University, Organization of African Unity St, POB: 11566, Cairo, Abbassia Egypt

**Keywords:** Solid state fermentation, Paromomycin, *Streptomyces rimosus*, Response surface methodology, Corn bran

## Abstract

**Background:**

Paromomycin is a 2-deoxystreptamine aminocyclitol aminoglycoside antibiotic with broad spectrum activity against Gram-negative, Gram-positive bacteria and many protozoa. This study introduces a strategy for paromomycin production through solid-state fermentation using *Streptomyces rimosus* subsp. *paromomycinus* NRRL 2455. Solid state fermentation has gained enormous attention in the development of several products because of their numerous advantages over submerged liquid fermentation. After selecting the best solid substrate, a time course study of paromomycin production was carried out followed by optimization of environmental conditions using response surface methodology. Paromomycin yields obtained using this technique were also compared to those obtained using submerged liquid fermentation.

**Results:**

Upon screening of 6 different substrates, maximum paromomycin concentration (0.51 mg/g initial dry solids) was obtained with the cost-effective agro-industrial byproduct, corn bran, impregnated with aminoglycoside production media. Optimization of environmental conditions using D-optimal design yielded a 4.3-fold enhancement in paromomycin concentration reaching 2.21 mg/g initial dry solids at a pH of 8.5, inoculum size of 5% v/w and a temperature of 30 °C.

**Conclusion:**

Compared to submerged liquid fermentation, solid state fermentation resulted in comparable paromomycin concentrations, cost reduction of raw materials, less energy consumption and waste water discharge, which have major implications in industrial fermentation. Therefore, solid state fermentation is a promising alternative to submerged liquid fermentation for paromomycin production. To the best of our knowledge, this is the first report on the optimized paromomycin production through solid state fermentation process.

## Background

Aminoglycosides are a set of antibiotics naturally produced from *Streptomyces* spp. or *Micromonospora* spp. [1, 2]. They possess activity against a wide range of microbes, including mycobacteria, protozoa, Gram-positive, Gram-negative bacteria and multiple drug-resistant pathogens [1, 3]. Of these aminoglycosides, paromomycin is a member of the therapeutically most relevant aminoglycosides, subclass 2-deoxystreptamine-aminocyclitol aminoglycoside antibiotics (2DOS-ACAGAs). It is active against Gram-negative and most Gram-positive bacteria, particularly *Staphylococcus* strains resistant to oxytetracycline, erythromycin or carbomycin [4]. Paromomycin also proved to be effective against many protozoal infections including visceral leishmaniasis, noninvasive amebiasis and giardiasis when other agents are contraindicated [5, 6]. It possesses the combination of high anti-amoebic and antibacterial activity with low oral toxicity, making it unique when compared to other available drugs [7].

Antibiotics have been generally produced by submerged liquid fermentation (SLF), and many approaches have been used to enhance their production [8, 9]. However, this technique is accompanied with some difficulties. For example, high-speed agitation for long incubation periods is usually required, which is accompanied by high energy consumption. In addition, the expensive substrates used, large volumes of broth and resulting wastewater, which must be treated, all share in increasing the costs of antibiotic production [10].

Solid state fermentation (SSF) is a biotechnological process where microorganisms are grown on solid substrates with no free water [11]. The substrate may simply be an inert supporting material or a nutrients’ source [12], including cereal grains, lignocellulose materials and a broad range of plant or animal materials. The unique interest in SSF is due to its relative simplicity, using plentiful cheap biomaterials with minimal pretreatment, low waste water production, and the ability to simulate comparable micro-environments, beneficial to microbial growth [13]. Other advantages include inexpensive production process, uncomplicated product recovery, and also reduced energy requirements for agitation and sterilization due to low amounts of water [14]. Different bio-products have been produced using SSF, including enzymes [15], biosurfactants [16], vitamins [17] and antibiotics [18, 19]. In recent years, researchers have shifted antibiotic production from SLF to SSF. For example, Asagbra et al. [20] used groundnut shell for the successful production of oxytetracycline from *Streptomyces* spp., while Vastrad and Neelagund [21] used oil pressed cake for the production of rifamycin B from *Amycolatopsis Mediterranei* MTCC 14.

For SSF to be successful, different factors like microorganisms, solid substrate, temperature and pH used should be considered. Moreover, the synthesis of antibiotics by *Streptomyces* spp. widely fluctuates according to the used culture conditions [22]. To obtain maximum production of antibiotics under SSF, it is imperative to optimize the environmental and nutritional factors. Response surface methodology (RSM), a combination of mathematical and statistical approaches for designing and analysis of complex processes, is commonly utilized to aid in the optimization process, with a rather small number of experiments and minimum effort [23]. It determines the effect of simultaneously varying different variables on the required response [24], explores the interactive effects between factors and avoids misusing precious time and supplies [25].

Paromomycin production, from *Streptomyces rimosus* (*S. rimosus*) *subsp. paromomycinus* NRRL 2455, has been optimized in our previously published report [9] under SLF conditions. Therefore, our aim was to optimize the nutritional and environmental conditions using RSM for maximum paromomycin production by the same strain, however, under SSF. Literature survey reveals, this is the first study on paromomycin production and optimization by *S. rimosus*, under SSF.

## Results

### Paromomycin production by SSF using different solid substrates

Out of the 6 solid substrates tested, corn bran and soybean meal resulted in the largest inhibition zones (22 ± 0.35 mm and 15.50 ± 0.71 mm, respectively). Therefore, a mixture of these 2 substrates was tested, and resulted in an IZ = 13.30 ± 0.42 mm. Therefore, corn bran, whose production corresponded to 0.51 mg/g IDS (0.34 mg/ml A6), was selected for further experiments.

### Factors affecting paromomycin production using SSF

#### Comparing time course of paromomycin production in SSF with production in SLF

**Fig. 1:**
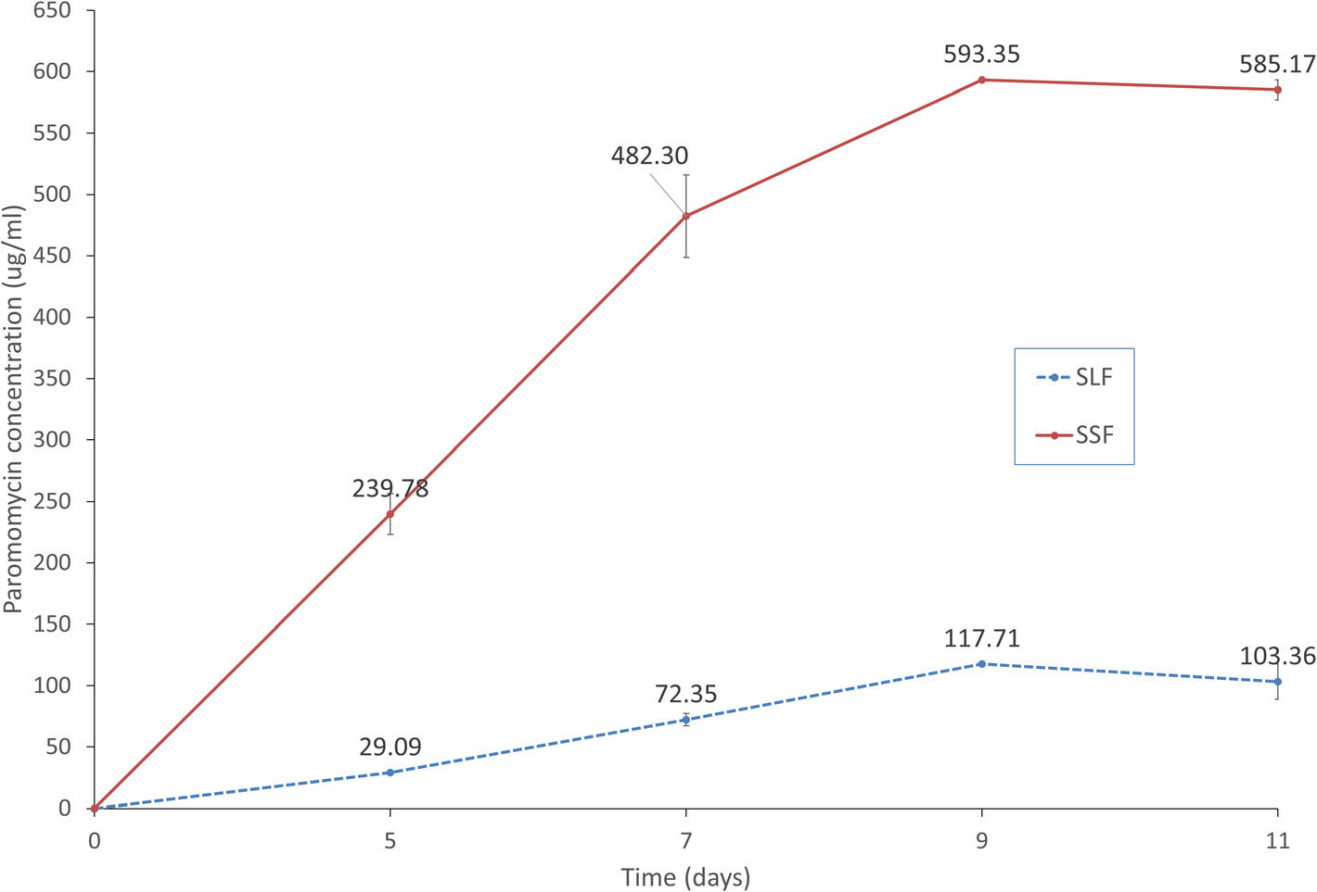
Time course of paromomycin production by *S. rimosus* NRRL 2455 under SSF and SLF

Figure 1 shows the time course of paromomycin production in both SSF and SLF. In SSF, production increased at the beginning to reach a concentration of 239.78 μg/ml A6 after 5 days of incubation. This concentration continued to rise reaching 593.35 μg/ml A6 after 9 days of incubation after which production remained nearly constant. Consequently, results in following experiments were attained after 9 days of incubation.

Using SLF, paromomycin production increased to reach 29.09 μg/ml at day 5 and 72.35 μg/ml at day 7. A maximum of 117.71 μg/ml was observed at day 9. Production slightly decreased upon further incubation.

#### Optimization of paromomycin production in SSF using RSM

The responses obtained after performing the 16 experiments proposed by the software were recorded in Table 1. From these results, the software automatically predicts a model which is a good fitting second-order polynomial equation relating the response with the tested factors. This predicted equation is then used by the software to calculate the predicted responses (Table 1) and build statistical and graphical summaries. This equation is given as follows:

$$ \mathrm{IZ}\ \left(\mathrm{mm}\right)=-36.38177+0.23237\ast \mathrm{A}+4.12160\ast \mathrm{B}-0.23124\ast \mathrm{C}+0.49451\ast \mathrm{A}\ast \mathrm{B}+6.47881\mathrm{E}-003\ast \mathrm{B}\ast \mathrm{C}-0.85128\ast {\mathrm{A}}^2-0.14051\ast {\mathrm{B}}^2 $$

**Table 1:** D-optimal design showing the experimental runs carried out and the observed and predicted responses

Run Order	pH (A)	Temperature (B, °C)	Inoculum size (C, % v/w)	IZ diameter (mm)	
				Observed response	Predicted response
1	8.50	37.00	5.00	20	19.79
2	8.50	23.00	5.00	21	20.82
3	5.50	37.00	5.00	0	0.04
4	8.50	37.00	35.00	21	20
5	8.50	23.00	35.00	18	18.35
6	6.25	30.00	12.50	23	21.26
7	5.50	37.00	35.00	0	0.2
8	8.50	37.00	5.00	19	19.79
9	7.00	30.00	20.00	24	23.83
10	5.50	23.00	20.00	21	20.52
11	7.00	30.00	35.00	22	23.27
12	5.50	23.00	5.00	21	21.75
13	8.50	23.00	35.00	19	18.35
14	5.50	37.00	35.00	0	0.21
15	7.00	23.00	5.00	23	23.2
16	8.50	30.00	20.00	26	26.64

As displayed in ANOVA results (Table 2), a Model F-value of 163.16 was obtained which confirms the significance of the model, since there is only a 0.01% probability that this large F-value could be caused by noise (*P*-value < 0.0001). Of the tested factors, pH (A) and temperature (B) were found to be significant since they had a P-value < 0.05 (Table 2). AB and B^2^ were also found to be significant model terms, while the rest of the terms were insignificant. The lack of fit P-value was 0.1299 and was statistically insignificant. In addition, a low coefficient of variation value (CV = 5.81%) was attained, which implies that the experimental results were reliable. The coefficient of determination, R^2^, was 0.993, meaning that our model can explicate 99.3% of the variability in response. The Predicted R-Squared (Pred R^2^ = 0.97) and Adjusted R-Squared (Adj R^2^ = 0.99) were in satisfactory agreement with each other. Finally, the Adequate precision ratio recorded (Adeq prec = 37.35) indicated a reasonable signal and that the current model may well be used to navigate the design space.

**Table 2:** ANOVA for Response Surface Reduced Quadratic Model for paromomycin production

Source	Sum of Squares	Degrees of freedom	Mean square	F-value	***P***-value
**Model**	1165.59	7	166.51	163.16	< 0.0001
**A-pH**	221.75	1	221.75	217.28	< 0.0001
**B-temperature**	273.06	1	273.06	267.56	< 0.0001
**C-inoculum size**	3.04	1	3.04	2.98	0.1226
**AB**	246.36	1	246.36	241.39	< 0.0001
**BC**	4.12	1	4.12	4.04	0.0794
**A**^**2**^	5.06	1	5.06	4.96	0.0565
**B**^**2**^	76.33	1	76.33	74.79	< 0.0001
**Residual**	8.16	8	1.02		
**Lack of fit**	7.16	5	1.43	4.30	0.1299
**Pure Error**	1.00	3	0.33		
**Corrected total**	1173.75	15			

**Fig. 2:**
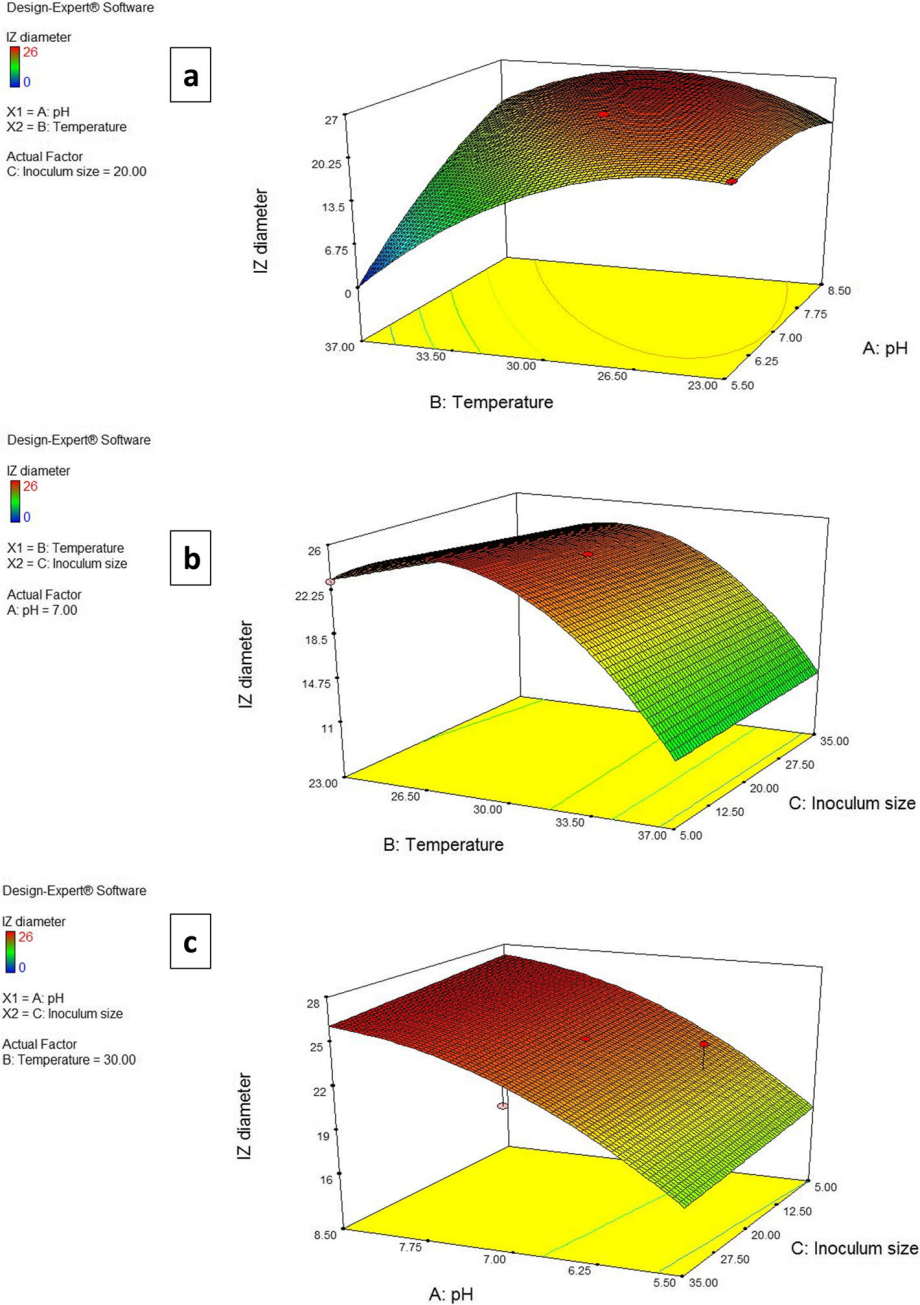
Three-dimensional surface plots representing the effect of 3 factors on inhibition zone diameter. When the effect of two parameters was plotted, the remaining one was set at central level **a** temperature and pH **b** temperature and inoculum size **c** pH and inoculum size

**Fig. 3:**
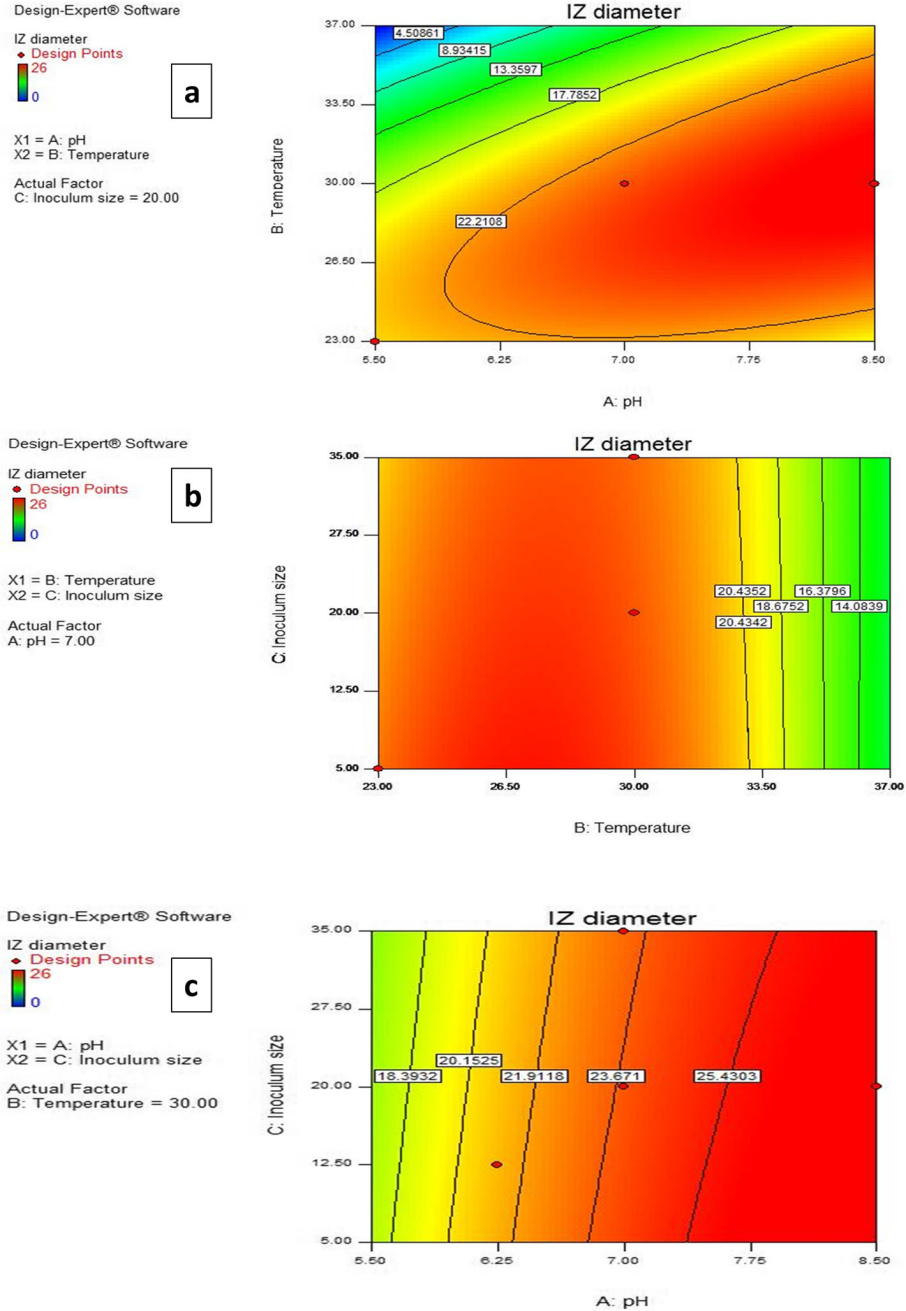
Contour plots representing the effect of 3 factors on inhibition zone diameter. When the effect of two parameters was plotted, the remaining one was set at central level **a** temperature and pH **b** temperature and inoculum size **c** pH and inoculum size

The three dimensional (3D) and contour plots between the factors are depicted in Figs. 2 and 3. Using these plots together with numerical optimization function in the Design expert software, the optimum conditions for maximum paromomycin production were suggested to be a pH of 8.5, temperature of 30 °C and inoculum size of 5% v/w .

**Model diagnostics:** to justify our model, 4 plots were constructed.

**Fig. 4:**
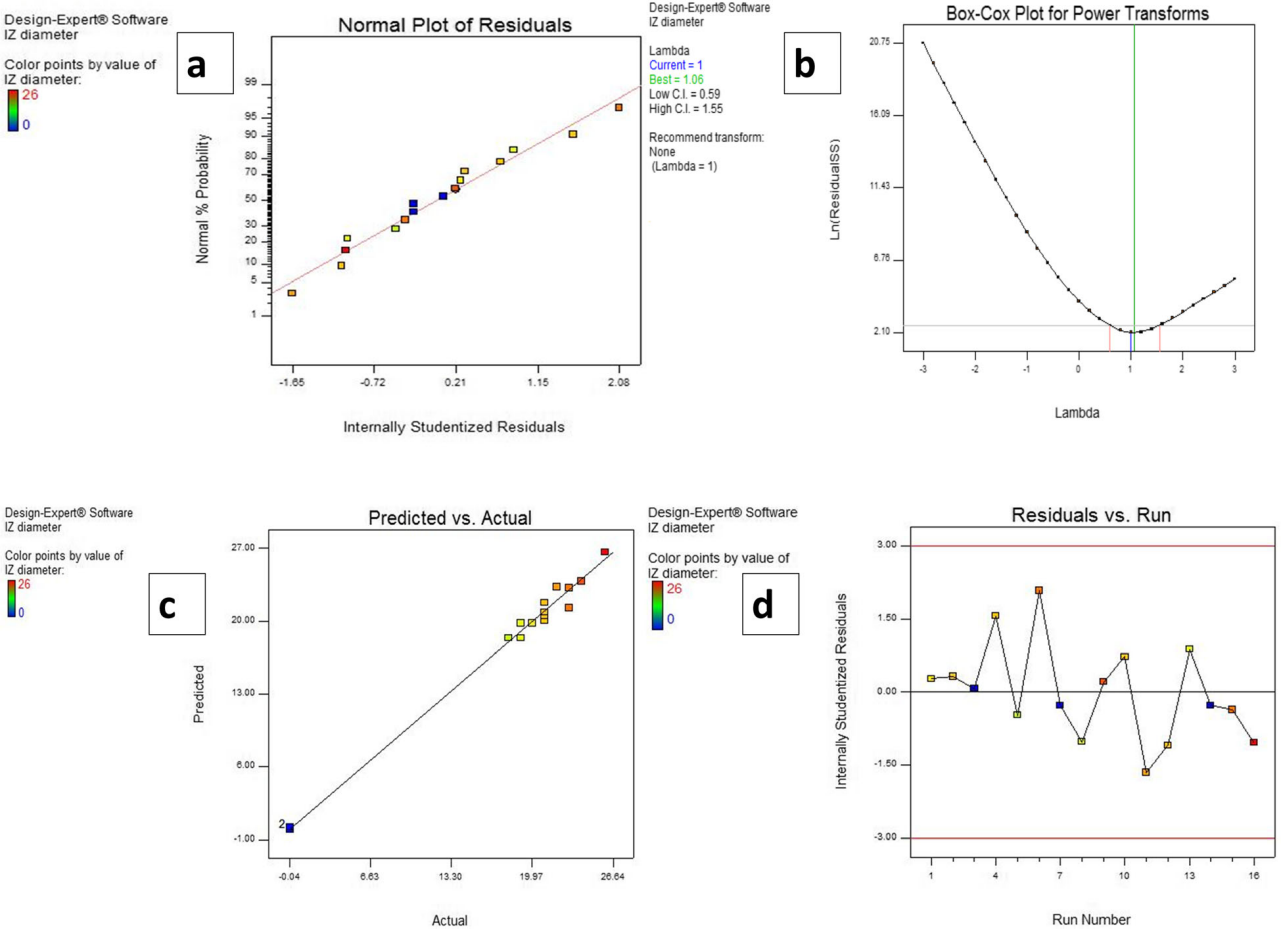
Model diagnostic plots **a** The normal probability plot of residuals, **b** Box Cox plot, **c** Predicted versus actual values plot and **d** Residuals versus Run number plot

**Normal probability plot** determines if the residuals follow a normal distribution. As shown in the figure, the points form a straight line, indicating a normal distribution of the residuals (Fig. 4a).

**Box Cox plot** is used for the determination of the best power transformation to be applied to response data. The Box–Cox plot indicated that no transformation was required which proves the sufficiency of the model (Fig. 4b).

**The predicted versus actual plot** the values in this plot were scattered near the straight line, suggesting that actual and predicted values were very similar (Fig. 4c).

**Residuals vs Run plot** plots the residuals against the experimental run order. It checks for lurking variables that may have affected the response during the runs (Design Expert Version 7 User’s Guide). Our plot displays the points scattered around zero implying the validity of the model (Fig. 4d).

### Experimental confirmation test

**Fig. 5:**
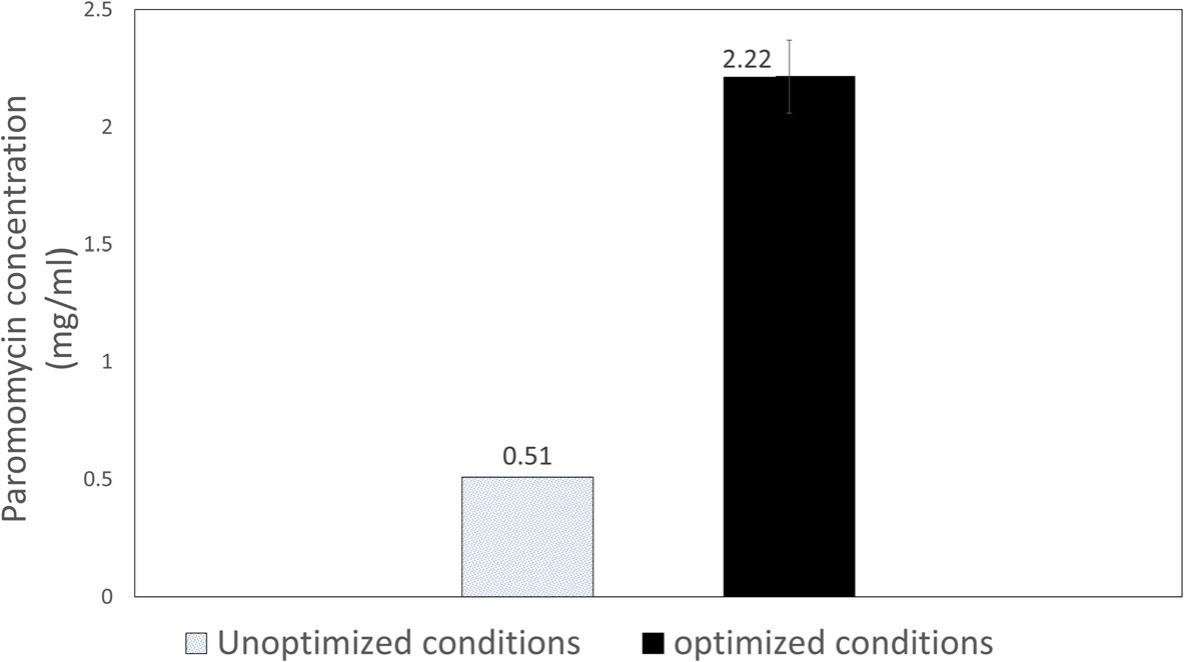
Comparison of paromomycin production by *S. rimosus* NRRL 2455 using optimized and unoptimized conditions

Using these suggested optimum values of the three factors, IZ diameter reached 27.25 ± 0.35 mm. This value was nearly identical to the predicted value (27.19 mm) which verifies the accuracy and practicality of RSM for optimization of fermentation processes. Using the calibration curve of standard paromomycin, we concluded that this inhibition zone corresponded to a concentration of 2.21 mg /g IDS. Hence, the optimal conditions used resulted in a 4.3-fold improvement in paromomycin production by *S. rimosus subsp. paromomycinus* NRRL 2455 when compared to that produced using unoptimized conditions (0.51 mg/g IDS) as shown in Fig. 5.

## Discussion

SSF has lately proven to be a fascinating alternative to SLF and has demonstrated consistency in numerous industries [13]. SSF is very successful in the synthesis of many novel antimicrobial agents, since its process conditions resemble the natural environment of *Streptomyces* sp. more closely than SLF [18]. Different bacterial species have been reported in literature to produce diverse antimicrobial agents under SSF using different solid substrates [18, 26, 27].

The present study was targeted at the optimization of the nutritional and environmental conditions for paromomycin production in SSF. Choosing a suitable substrate is a crucial aspect of SSF since it represents both a nutrients source and a physical support [14]. Substrate dependent bacterial product yield differences have been shown in previous studies and hence screening of several substrates is necessary [28]. Therefore, six different substrates were screened for paromomycin production by *S. rimosus* NRRL 2455. As shown in the results, the highest production was obtained using corn bran as the solid substrate. Corn bran is the most abundant and low-value agro-industrial byproduct of the milling process of corn [29, 30]. After corn processing, its bran is generally discarded or used as animal feed [31]. Corn bran is rich in carbohydrates (78%), proteins (3.5%), iron (16%) and fats (1%) [32]. It has been successfully used in SSF of many metabolites including biosurfactants [33], enzymes [32] and antibiotics [26]. Using agro-industrial by-products as carbon and energy sources is advantageous for two reasons: the use of a cheap substrate and a fascinating way of adding worth to a by-product [34]. Hence, using this inexpensive agricultural residue will drastically reduce the costs of paromomycin production, and will also tile the way to efficient managing of solid wastes.

Production of antibiotics is significantly improved by the supplementation of numerous carbon and nitrogen sources in fermentation medium [35]. Results from our previous study showed that aminoglycoside production medium (A6), consisting of glycerol and CaCO_3_ as carbon sources and soybean meal and NH_4_Cl as nitrogen sources, was the optimum medium for paromomycin production in SLF since it resulted in the highest specific productivity [9]. Therefore, A6 media was selected in this study as the impregnating solution. Similar results were obtained in previous studies, where 1% w/w CaCO_3_ enhanced the tetracycline production in SSF using *S. rimosus* [36]. Moreover, NH_4_Cl positively enhanced neomycin production [37], while soybean meal enhanced rifamycin B concentrations by *Amycolatopsis* sp. RSP 3 under SSF [35]. The volume of impregnating solution used depended on the substrate’s liquid absorption capacity, which was different for each substrate. The absorption capacity is defined as the volume that can be added to 10 g of dry substrate without the emergence of free liquid [33]. This was to ensure that optimum moisture levels were used, and to avoid excess moisture which might negatively affect antibiotic production.

After determining the best substrate, it was necessary to perform a kinetic study to investigate the best incubation time required for maximum paromomycin production. A short incubation period may result in incomplete antibiotic formation while excess incubation may cause nutrient exhaustion and accumulation of toxic metabolites which hinders further increase in antibiotic production [26]. As depicted in the results, a maximum concentration of 593.35 μg/ml was obtained after 9 days of incubation. Therefore, extraction in ensuing runs was carried out at this time. Various researches have recorded different optimum incubation periods. Remarkably high levels of neomycin were achieved at the 10th day of fermentation in one study [38]. Maximum rifamycin SV production was obtained on day 9, followed by a steady decline in production [26]. Recently, maximum antibiotics concentration by *Streptomyces* sp. was achieved after 8 days of incubation [18].

A time course of paromomycin production under SLF using the impregnating solution as production media (A6 media) and the same cultivation conditions was also carried out to compare the production obtained with that obtained under SSF. As shown in the results, best incubation time was also found to be 9 days, however, a 5-fold improvement in production was observed in case of SSF, which highlights the superiority of this process. Similar results were obtained by Mahalaxmi et al. [35] who noticed that maximum rifamycin B production was obtained after 9 days of incubation under both SSF and SLF with *Amycolatopsis* sp. RSP 3, however, values were 4-fold greater in SSF with corn husk. In addition, Tabaraie et al. reported that higher levels of cephalosporin were obtained by *A. chrysogenum* in SSF than in SLF [39].

Upon reviewing literature on SSF for antibiotic production, it was found that temperature, pH and inoculum size were some of the factors having a strong impact on SSF [40]. Therefore, these 3 factors were optimized using D-optimal experimental design in RSM. RSM is an efficient technique that can determine the best fermentation conditions for a multi-variable system mathematically and statistically [41]. It is favorable over classical optimization methods because it is fast, reliable, helps understand the effect of varying concentrations of nutrients and leads to a substantial reduction in total number of runs therefore saving time, chemicals and manpower [42]. RSM has been considerably used for the optimized production of many antibiotics [18, 37]. This technique comes with numerous types of designs for the optimization of important fermentation parameters, and D-optimal design is one of the most accurate ones [43]. It has been used by many investigators in optimization studies [44, 45].

A total of 16 runs were conducted to study how the 3 factors influenced paromomycin production. To investigate the significance of the design, we used ANOVA which provides a better understanding of the sources of variation and is the most exploited statistical tool to evaluate the variables’ impact over a process [35]. The attained F-value (163.16) proved that the model developed in this study was significant and may be used to explain paromomycin production by SSF. Alternatively, the lack of fitness should be non-significant for the model to fit well with the experimental design. In our results, the non-significant lack of fit (*P*-value = 0.1299) showed that the model was appropriate for the current study. The CV indicates the precision level with which the treatments are compared, and model reliability usually declines as the CV value rises [46]. Our low CV indicates adequate reliability of the experimental values. In addition, the obtained R^2^ demonstrates a close agreement between the experimental and the predicted values. The ability of the model to precisely predict a response value can be expressed as the predicted R^2^, which should be in good agreement with the adjusted R^2^, and difference between both shouldn’t exceed 0.2 [47]. In our study, the Pred R^2^ and Adj R^2^ were in fair agreement with each other. Adequate (Adeq) Precision assesses the signal to noise ratio, and a ratio greater than 4 is usually desirable [48]. Our Adeq precision ratio of 37.35 implied a satisfactory signal and that the model may be used to navigate the design space and may effectively be used to explain paromomycin production by SSF with *S. rimosus*.

For a better understanding of the variables’ effects on paromomycin production, the predicted model was presented as 3D plots. The 3D plots can directly reflect the effect of different levels of the factors on the response and therefore pinpoint their optimum levels [46]. Using these plots together with numerical optimization function, optimum conditions for highest production were found to be a pH of 8.5, temperature of 30 °C and inoculum size of 5% v/w, yielding a maximum IZ diameter of 27.25 mm which was nearly equal to the value predicted by the model proving the soundness of the model. The constructed model diagnostic plots further verified the validity of the model built.

*P*-value was also used to evaluate the significance of each of the tested variables. The smaller the P-value and larger the sum of squares, the more significant the corresponding factor is [49]. Results revealed that the factors A (pH) and B (temperature) had a significant effect on paromomycin concentration (*P* < 0.0001), temperature being more significant. However, factor C (inoculum size) had the least effect on paromomycin production. Similar findings were reported by Mahalaxmi et al. [35] who showed that inoculum level exhibited the least influence on antibiotic production. Temperature and pH are vital physiological parameters influencing the metabolic pathways, hence the generation of various metabolites [40]. In our study, maximum paromomycin concentration was obtained using pH 8.5. A pH 8.5 was also optimum for the maximum production of rifamycin B in SSF in a previous study [50]. On the other hand, maximum rifamycin SV production was obtained at pH 7 in a previous study [26]. This indicates that the best pH for maximum antibiotic production was strain dependent.

Another critical factor affecting antibiotic production is temperature. As depicted in the results, optimum temperature was found to be 30 °C. This is similar to results obtained in our previous study, where optimal temperature for highest paromomycin production under SLF was found to be 28 °C [9]. Results by other researchers were variable. Some studies showed that 30 °C was ideal for antibiotic production under SSF including neomycin [38], rifamycin SV [26] and cephalosporin C [51]. Others reported that maximum rifamycin B production in SSF by *A. mediterranei* strain MTCC 14 was obtained at 32 °C [21]. In our study, a decrease in paromomycin concentration was detected when the incubation temperature was lower than the optimum temperature. It has been reported by several researchers that low temperatures tend to slow down the metabolic activities of the microorganisms [51]. Moreover, production was completely abolished at a temperature of 37 °C in acidic or neutral conditions. This may be because heat evolved during SSF process is poorly dissipated and therefore gets accumulated in the medium, resulting in decreased microbial activity and growth, thus reducing the product yield [26].

Moreover, ANOVA results revealed that the model term AB was significant, meaning that the interaction between pH and temperature was significant, while the other interaction terms were insignificant. A significant interaction between 2 factors means the effect of one factor is dependent on the level of the other [52]. Contour plots can reveal the significance of the interaction between two factors: an elliptical contour implies a significant interaction between the two factors, whereas a circular contour implies that the interaction between the two factors is weak [49]. As depicted in the results, the contour plot obtained for AB was oval in shape indicating significant interaction between these variables.

Therefore, optimization of paromomycin production by D-optimal design resulted in a maximum IZ diameter of 27.25 mm which was equivalent to 2.21 mg /g IDS or 1.47 mg/ml A6. Therefore, a 4.3-fold enhancement in production was attained in comparison to production obtained using unoptimized conditions.

It is interesting to note that in our previous study, paromomycin production was optimized under SLF, resulting in a maximum paromomycin concentration of 1.58 mg/ml A6 [9]. Upon comparing these results with results obtained in the current study using SSF, it was found that comparable antibiotic levels were obtained using SSF in nearly the same period of time as SLF (9 days) and nearly the same inoculum size, however, using cheaper substrates and a relatively simpler technique. In addition, *Streptomyces* mycelium morphology is well-matched to invasive growth on solid culture. This morphology accounts for substantial problems in SLF, including sheer forces, increased viscosity due to the metabolic secretion, and a reduced metabolic stability, which leads to very high mixing requirements and oxygen transfer efficiency in addition to product recovery complications [53]. Therefore, SSF process may be utilized as a substitute, permitting better oxygen circulation, less waste water production and reduced energy requirements for stirring and sterilization making it a more attractive technique for paromomycin production.

## Conclusion

This study proposed a SSF strategy for paromomycin production by *S. rimosus* NRRL 2455 utilizing corn bran as an excellent however cheap substrate. DOD and RSM were efficacious in improving paromomycin production under SSF by 4.3 folds and a maximum concentration of 2.21 mg/g IDS was attained in the present study after 9 days of incubation. Optimum fermentation conditions were recorded to be a pH of 8.5, an inoculum size of 5% v/w and a temperature of 30 °C. SSF showed remarkable advantages in terms of cost reduction of raw materials, less energy consumption and no waste water discharge. These results suggested that SSF is a better alternative to produce paromomycin since our maximum concentration was comparable to the values yielded in SLF under optimized conditions. Thus, *S. rimosus* NRRL 2455 can be regarded as a promising bacterial strain and additional studies to the application of SSF for paromomycin production and moving up to industrial scale is therefore vindicated.

## Methods

### Microorganisms

*S. rimosus* subsp. *paromomycinus* NRRL 2455 (paromomycin producer; kindly provided by NRRL, USA) was maintained on Trypticase soy agar (TSA) plates, sub cultured every month and preserved at 4 °C. For long term preservation, it was kept in Trypticase soy broth (TSB) containing 50% glycerol at − 80 °C [54]. The standard strain, *Staphylococcus aureus* ATCC 25923, was used for the bioassay of paromomycin produced by *S. rimosus*.

### Culture media

TSB was the seed culture media used for development of *S. rimosus* subsp. *paromomycinus*. Aminoglycoside production media (A6 media) [55] was also prepared and used in this study and was composed of (g/L): soybean meal 30, CaCO_3_ 5, NH_4_Cl 4 and glycerol 40 ml/L and distilled water to 1 L. The media pH was initially adjusted to 7.

### Production of paromomycin

#### Seed culture

*S. rimosus* was streaked on TSA plates and incubated for 72 h at 28 °C. An isolated colony was then inoculated in a 250 ml flask holding 25 ml TSB and incubated at 28 °C and 200 rpm for 48 h.

#### Paromomycin production by SSF

Each 250 ml Erlenmeyer flask contained ten grams of either sugarcane bagasse (residual after withdrawal of the juice from the sugarcane stalks attained at a local market, cut into little pieces), sunflower seed meal (sunflower seeds ground and sieved through a 1.4 mm mesh sieve), soybean meal, barley, corn bran or wheat bran (all obtained from a local market) after drying at room temperature (Table 3). A flask containing a mixture of corn bran and soybean meal (5 g each) was also prepared. After sterilization by autoclaving (15 min at 121 °C), the flasks were moistened with A6 media (volume added according to the substrate’s liquid absorption capacity (see Table 3)) [33] inoculated with 2 ml of seed culture (1 × 10^7^ cfu/ml) which was equivalent to 20% v/w. Two milliliters of seed culture resulted in a final bacterial concentration = 2 × 10^6^ cfu/g solid substrate [38, 53]. Growth in the seed culture was determined using the viable count technique [56]. The tested flasks were subsequently incubated for 6 days at 30 °C under static conditions. Control flasks consisted of the different substrates treated similarly but without inoculation.

**Table 3:** Solid substrates screened and their liquid absorption capacity (ml) of A6 media

Solid substrate	Liquid absorption capacity (ml) of A6 media per 10 g of solid substrate
Sugarcane bagasse	25
Corn bran	15
Sunflower seed meal	15
Soybean meal	20
Wheat bran	15
Barley	20
Corn bran + soybean meal	20

### Extraction of paromomycin

At the end of the incubation period, 50 ml of distilled water were added to each flask followed by agitation for 30 min at 30 °C and 200 rpm [57]. The resulting suspensions were passed through gauze pieces. The whole process was repeated twice. To obtain the supernatants, the filtrates were then pooled and centrifuged (10 min at 10,000 rpm) [37, 51].

### Determination of the antimicrobial activity

To test the antibacterial activity of the produced paromomycin, the supernatants obtained after extraction were sterilized by filtration using 0.22 μm membrane filters (Ministart® syringe filter). Agar well diffusion technique was used to bioassay the culture filtrates against *Staphylococcus aureus* ATCC 25923 [53, 58]. Briefly, *Staphylococcus aureus* ATCC 25923 (a suspension equivalent to 0.5 McFarland) was homogenously spread on the surface of Mueller Hinton agar (MHA, Difco, USA). Ten mm wells were filled with 150 μl of the culture filtrate. After overnight incubation at 37 °C, the inhibition zone diameters (IZ mm) were recorded. The solid substrate that resulted in the largest inhibition zone was selected for further studies.

### Estimation of paromomycin

Paromomycin concentrations were calculated using the linear equation attained from the calibration curve constructed between standard paromomycin concentrations and IZ diameter in our previous study [9]:

$$ \mathrm{Y}\ \left(\log\ \mathrm{paromomycin}\ \mathrm{concentration}\ \mathrm{in}\ \mu \mathrm{g}/\mathrm{ml}\right)=0.1214\ \mathrm{X}-0.9642 $$

where X is IZ diameter (mm).

Paromomycin concentrations were expressed as the mg per gram of initial dry solids (mg/g IDS). Additionally, concentrations were expressed as milligrams per milliliter of A6 media added to the solid substrate (mg/ml A6) to compare SSF results with those obtained in SLF [33].

### Studying the different factors affecting paromomycin production using SSF

#### Studying the time course of paromomycin production in SSF using the selected substrate (corn bran)

Five flasks containing 10 g of the selected solid substrate were prepared. Fifteen milliliters A6 media inoculated with 2 ml of seed culture (13.33% v/v or 20% v/w) were added to the solid substrate followed by incubation at 30 °C. At specific time intervals, one flask was extracted for quantification of paromomycin concentration, over an incubation period of 11 days.

### Paromomycin production in SLF

SLF was also carried out to compare its production with production using SSF. Conditions used in this experiment were similar to conditions mentioned for SSF. Briefly, 25 ml of A6 media in 250 ml Erlenmeyer flasks were inoculated with the seed culture prepared (13.33% v/v) and incubated at 200 rpm and 30 °C. At specific time intervals, samples were withdrawn from the culture broth for extraction and determination of paromomycin concentration as mentioned above.

### Response surface methodology (RSM) for optimization of paromomycin production in SSF

The 3 factors, pH (A), temperature (B) and inoculum size (C), were optimized using RSM. Experimental D-optimal design (DOD) was chosen and the levels of the factors used were listed in Table 4. A total of 16 experiments were designed and carried out with 1 center point. At the end of each experiment, paromomycin was extracted and its antimicrobial activity was determined as explained above. One response value, IZ diameter (mm), was measured after 9 days of incubation. A second-order polynomial equation, which admits all interaction terms, was derived from the software and used to calculate the predicted response. The design of experiments was done by Design Expert® v. 7.0 (Design Expert® Software, Stat-Ease Inc., Statistics Made Easy, Minneapolis, MN, USA). Analysis of variance (ANOVA) was employed to validate the obtained model. The model significance was determined using F-test and a *P*-value < 0.05 was regarded as significant.

**Table 4:** Values of coded levels of the 3 tested factors used for the D-optimal design

Factor	Level		
	−1	0	+ 1
**pH (A)**	**5.5**	**7**	**8.5**
**Temperature (B, °C)**	**23**	**30**	**37**
**Inoculum size (C, % v/w)**	**5**	**20**	**35**

### Experimental verification of RSM results

Optimum culture conditions were predicted using the numerical optimization function in the Design Expert software and a new run employing these optimal factors was carried out to verify the model. Results obtained were compared with the value predicted by the model and with the paromomycin concentration yielded using unoptimized conditions.

### Statistical and graphical analysis

All experiments were performed in triplicate and the values calculated and plotted are the means of triplicate results while error bars indicate the standard deviation of the data. For RSM, all the experiments were carried out in triplicate and the average of three readings was recorded. Design of experiments, response surfaces, contour plots, model diagnostic plots and ANOVA analysis were obtained from Design Expert v. 7.0.

## Data Availability

All data generated or analyzed during this study are included in this published article.

## Abbreviations

Adj R^2^: Adjusted R^2^
Pred R^2^: Predicted R^2^
SLF: Submerged liquid fermentation
SSF: Solid state fermentation
IDS: Initial dry solids
DOD: D-optimal design
IZ: Inhibition zone
CV: Coefficient of variation
RSM: Response surface methodology
